# ANARCI: antigen receptor numbering and receptor classification

**DOI:** 10.1093/bioinformatics/btv552

**Published:** 2015-09-30

**Authors:** James Dunbar, Charlotte M. Deane

**Affiliations:** Department of Statistics, Oxford University, Oxford, UK

## Abstract

**Motivation:** Antibody amino-acid sequences can be numbered to identify equivalent positions. Such annotations are valuable for antibody sequence comparison, protein structure modelling and engineering. Multiple different numbering schemes exist, they vary in the nomenclature they use to annotate residue positions, their definitions of position equivalence and their popularity within different scientific disciplines. However, currently no publicly available software exists that can apply all the most widely used schemes or for which an executable can be obtained under an open license.

**Results:** ANARCI is a tool to classify and number antibody and T-cell receptor amino-acid variable domain sequences. It can annotate sequences with the five most popular numbering schemes: Kabat, Chothia, Enhanced Chothia, IMGT and AHo.

**Availability and implementation:** ANARCI is available for download under GPLv3 license at opig.stats.ox.ac.uk/webapps/anarci. A web-interface to the program is available at the same address.

**Contact:** deane@stats.ox.ac.uk

## 1 Introduction

The variable domains of antibodies and T-cell receptors (TCR) contain these proteins’ major binding regions. Alignment of these variable sequences to a numbering scheme allows equivalent residue positions to be annotated and for different molecules to be compared. Performing numbering is fundamental for immunoinformatics analysis and rational engineering of therapeutic molecules (Shirai, 2014).

Several numbering schemes have been proposed, each is favoured by scientists in different immunological disciplines. The Kabat scheme (Kabat *et al.,* 1991) was developed based on the location of regions of high sequence variation between sequences of the same domain type. It numbers antibody heavy (VH) and light (Vλ and Vκ) variable domains differently. Chothia’s scheme (Al-Lazikani, 1997) is the same as Kabat’s but corrects where an insertion is annotated around the first VH complementarity determining region (CDR) so that it corresponds to a structural loop. Similarly, the Enhanced Chothia scheme (Abhinandan and Martin, 2008) makes further structural corrections of indel positions.

In contrast to these Kabat-like schemes, IMGT (Lefranc, 2003) and AHo (Honegger and Plückthun, 2001) both define unique schemes for antibody and T cell receptor (TCR) (Vα and Vβ) variable domains. Thus, equivalent residue positions can easily be compared between domain types. IMGT and AHo differ in the number of positions they annotate (128 and 149 respectively) and where they consider indels to occur.

Separate online interfaces exist that can apply each numbering scheme: Kabat, Chothia and Enhanced Chothia through Abnum (Abhinandan and Martin, 2008); IMGT through DomainGapAlign (Ehrenmann, 2010); and AHo through PyIgClassify (Adolf-Bryfogle *et al*., 2015). No program currently exists that can apply all schemes or for which an executable is available under open license.

We have developed ANARCI, a program that can annotate sequences with all five of the numbering schemes described above. We provide both a web-interface and the software under open license so that these fundamental annotations can be easily available for further immunoinformatics analyses.

## 2 Algorithm

ANARCI takes single or multiple amino-acid protein sequences as input. The program aligns each sequence to a set of Hidden Markov Models (HMMs) using HMMER3 (Eddy, 2009). Each HMM describes the putative germ-line sequences for a domain type (VH, Vλ or Vκ, Vα or Vβ) of a particular species (Human, Mouse, Rat, Rabbit, Pig or Rhesus Monkey). The most significant alignment is then used to apply one of five numbering schemes.

### 2.1 Building Hidden Markov Models

The HMM for each domain type from each species was built in the following way:

1. The pre-aligned (gapped) germ-line sequences for the v-gene segment of each available species and domain type were downloaded from the IMGT/Gene Database (Giudicelli, 2005). The sequences of the j-gene segment were also downloaded. These were aligned to a single reference sequence using Muscle (Edgar, 2004) with a large (−10) gap-open penalty.
2. All possible pairwise combinations of the relevant v and j gene segments were taken to form a set of putative germ-line domain sequences. For the VH domain, the d gene segment was not included. Each position in the alignment represents one of the 128 positions in the IMGT numbering scheme.
3. From the alignment an HMM is built using the hmmbuild tool. Here, the ‘—hand’ option is specified to preserve the structure of the alignment.

In total, 24 HMMs were built describing variable domain types from six different species. These HMMs were combined into a single HMM database using hmmpress.

### 2.2 Numbering an input sequence

An input sequence is aligned to each HMM using hmmscan. If an alignment has a bit-score of less than 100 it is not considered further. This threshold proves effective at preventing the false recognition of other IG-like proteins. Otherwise, the most significant alignment classifies its domain type and the alignment is translated into a chosen numbering scheme.

ANARCI can apply the Kabat, Chothia, Extended Chothia, IMGT or AHo schemes to VH, Vλ and Vκ domain sequences. The IMGT and AHo schemes can also be applied to Vα and Vβ domain sequences. Where possible, a position in the HMM alignment is annotated with the equivalent position in the numbering scheme. In regions where there is no direct equivalence between the alignment and the numbering scheme the sequence is numbered according to the specification described in the corresponding publication. For example, HMM alignment position 40 for a VH sequence is equivalent to Kabat position 31-35X depending on the length of CDRH1.

For each numbered domain a header is written that describes the most significant alignment including the species, chain type and alignment range. The numbering follows in a column delimited format. Alternatively, users may import ANARCI as a Python module and use the API within their own scripts.

## 3 Benchmark

With the rise of next generation sequencing, the ability to annotate large numbers of antibody sequences is becoming a common task. We used ANARCI to number a set of 1 936 119 VH sequences taken from a vaccination response study performed at Oxford University. The algorithm took three hours wall clock time using 32-cores with AMD Opteron 6272 Processors. All but 9560 sequences were successfully numbered. Where numbering failed the sequences had very unusual insertions or deletions that may be a result of sequencing errors.

## 4 Webserver

**Fig. 1.**
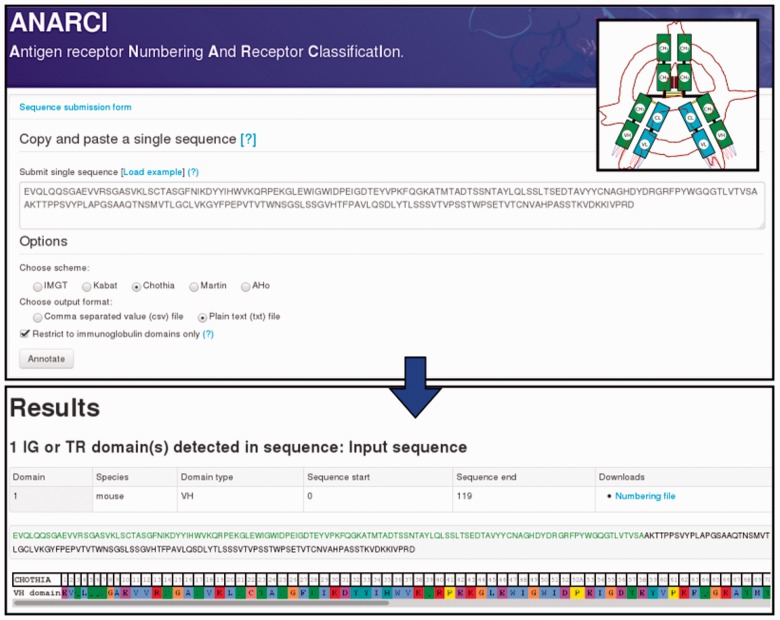
The web-based interface to ANARCI. The species, domain type and numbering is reported for each sequence. The annotations can either be downloaded or visualized on the webpage

In addition to the command line tool we provide a webserver interface to ANARCI (Fig. 1). Users may submit a single amino-acid sequence or a Fasta file of multiple sequences to apply their chosen scheme. The interface displays the assigned species and type of domain, the location of each domain in the sequence and, using the JSAV library (Martin, 2014), the annotated numbering scheme. Plain text or CSV formatted output files are available for download.

## 5 Conclusion

We have developed ANARCI, a program for annotating antigen receptor variable domain amino-acid sequences with five commonly used numbering schemes. The program can be run as command-line tool or imported as a Python module for incorporation in custom scripts. We also provide a public web-browser interface that can annotate small numbers of sequences. ANARCI is freely distributed under the GPLv3 license and available at opig.stats.ox.ac.uk/webapps/anarci.
